# In Vitro Fertilization Using Luteinizing Hormone-Releasing Hormone Injections Resulted in Healthy Triplets without Increased Attack Rates in a Hereditary Angioedema Case

**DOI:** 10.1155/2018/2706751

**Published:** 2018-02-13

**Authors:** Ceyda Tunakan Dalgıç, Fatma Düşünür Günsen, Gökten Bulut, Emine Nihal Mete Gökmen, Aytül Zerrin Sin

**Affiliations:** Department of Internal Medicine, Division of Allergy and Clinical Immunology, Ege University Medical Faculty, İzmir, Turkey

## Abstract

Hereditary angioedema due to C1-inhibitor deficiency (C1-INH-HAE) is a rare, autosomal dominant disorder. The management of pregnant patients with C1-INH-HAE is a challenge for the physician. Intravenous plasma-derived nanofiltered C1-INH (pdC1INH) is the only recommended option throughout pregnancy, postpartum, and breastfeeding period. In order to increase pregnancy rates, physicians use fertilization therapies increasing endogen levels of estrogens. Therefore, these techniques can provoke an increase in the number and severity of edema attacks in C1-INH-HAE. Our patient is a 32-year-old female, diagnosed with C1-INH-HAE type 1 since 2004. She had been taking danazol 50–200 mg/day for 9 years. Due to her pregnancy plans in 2013, danazol was discontinued. PdC1INH was prescribed regularly for prophylactic purpose. Triplet pregnancy occurred by* in vitro* fertilization using luteinizing hormone-releasing hormone (LHRH) injections. In our patient, LHRH injections were done four times without causing any severe attack during* in vitro* fertilization. Angioedema did not worsen during pregnancy and delivery due to the prophylactic use of intravenous pdC1INH in our patient. According to the attack frequency and severity, there was no difference between the three pregnancy trimesters. To our knowledge, this is the first published case of C1-INH-HAE receiving* in vitro* fertilization therapies without any angioedema attacks during pregnancy and delivery and eventually having healthy triplets with the prophylactic use of intravenous pdC1INH.

## 1. Introduction

C1-INH-HAE is characterized by recurrent attacks of edema formation in the skin, upper airways, and the gastrointestinal tract [1]. Three types of hereditary angioedema (HAE) have been described: two of them are due to C1 inhibitor (C1-INH) deficiency (C1-INH-HAE types I and II) and one is characterized by normal C1-INH (nC1-INH-HAE). The management of pregnant patients with C1-INH-HAE is often a challenge, because their symptoms can become more prevalent because of elevated estrogen levels. Attenuated androgens, such as danazol, are contraindicated in pregnancy and breastfeeding [2]. Surgical procedures (such as cesarean section) are associated with an increased risk of attacks, and continuing postpartum attacks can prevent mothers from breastfeeding [2–5]. Nanofiltered plasma-derived C1-INH concentrate (pdC1INH) is the only therapeutic option recommended throughout pregnancy, the postpartum period, and breastfeeding [2].

## 2. Case Presentation

A 32-year-old female was diagnosed with hereditary angioedema type 1 (C1-INH-HAE) in 2004. Her major symptoms were abdominal and peripheral edema attacks. She had been taking danazol (dosage: 50–200 mg/day) for 9 years due to the frequency and severity of the attacks. She had abdominal attacks every month before her menstrual cycle; breakthrough attacks had been effectively controlled with pdC1INH (Cetor®, 500 IU, Sanquin, Amsterdam, Netherlands). Due to her pregnancy plans in 2013, danazol was stopped 1 year before the conception attempts. After changing therapy, angioedema attacks started again; so prophylactic intravenous pdC1-INH was administered (dosage: 1000 U) twice a week. Between 2013 and 2014,* in vitro* fertilization therapies started. Intravenous pdC1INH 1000 U twice a week was continued and during that period she received LHRH injection four times. Prior to every LHRH injection, an extra dosage of 1000 U intravenous pdC1INH was administered. Although plasma estrogenic status increased during* in vitro* fertilization therapies, no attacks were observed during LHRH injections by means of extra dosages of pdC1INH. Triplet pregnancy was achieved by* in vitro* fertilization methods in 2014. Prophylaxis with pdC1INH 1000 U every 2 days was prescribed during her pregnancy until the 20th week. After cerclage operation at the 20th week, prophylactic intravenous pdC1INH was increased up to 1500 U every 2 days because of weight gain. Although she had gained extra 20 kg weight, she did not have any angioedema attacks probably due to the prophylactic procedure. Before and 12 hours after cesarean section, extra 1000 U of intravenous pdC1INH was administered. Cesarean section was performed under epidural anesthesia at the 32nd week of the gestation with uneventful delivery. Three healthy babies (1 female and 2 males) were born without any complications. The patient received 188 vials (94.000 units) of pdC1INH in total during her whole pregnancy, which successfully prevented the recurrence of attacks. After the delivery, because of breastfeeding, intravenous pdC1INH was continued 1000 U twice a week regularly (Table 1). The patient suffered 2 abdominal attacks during the 4 months of breastfeeding, which were treated with extra dosages of 1000 U pdC1INH for each attack. Those attacks were thought to be related to uterine contractions because of hormonal changes during lactation. She received in total 64 vials (32.000 units) of intravenous pdC1INH during breastfeeding. No side effects were observed. Prior to and after completion of pregnancy and breastfeeding, hepatitis A, B, C viruses (HAV, HBV, and HCV), human immune deficiency virus (HIV), Epstein-Barr virus (EBV), cytomegalovirus (CMV), and parvovirus B19 were tested and the tests resulted negative. Viral transmission did not occur as a result of pdC1INH therapy. No antibodies against pdC1INH were either detected. Repeated administration did not reduce the efficacy of pdC1INH.

## 3. Discussion

Women with C1-INH-HAE suffer from more frequent and more severe attacks than men [2]. The disease is often affected by estrogenic status due to increased kininogenase activities [5, 6]. An awareness of the disease is critical for clinicians involved in the care of women because of potential complications related to pregnancy, labor, and delivery and other women's health issues [7]. Pregnancy aggravates symptoms in 40% of cases, and exogenous estrogen often aggravates the disease [8]. Some women report that their symptoms coincide with their periods [8]. Estroprogestin contraception increases the frequency of attacks in 63% to 80% of women, and hormone replacement treatment can trigger the appearance of the first symptoms [8]. Deliveries seem to be safe but it is advised to have pdC1INH available in the delivery room; in case of worsening during the pregnancy, it is recommended to use short term prophylaxis with intravenous pdC1INH [8]. As shown in the literature, our patient had the risk of increased attack frequency, especially because of estrogenic therapies (like LHRH injections) [9]. There are many treatment schedules in which LHRH agonists are used in assisted reproductive technology. Their duration and initiation particularly in ovarian hyperstimulation* in vitro* fertilization (IVF) injection treatments vary. Pulsatile administration of LHRH in physiologic amounts at a frequency that is similar to the endogenous release stimulates the ovaries which will induce ovulation [9]. Also, significantly higher serum estradiol concentration and increased number of oocytes are observed in IVF treatments due to the cyclic supplementation of LHRH [10]. However, due to long term prophylaxis and short term prophylaxis with pdC1INH, our patient did not suffer from angioedema attacks. For the treatment of our patient, we had to administer very high therapeutic dosages of pdC1INH: in total 126000 units during pregnancy, delivery, and breastfeeding. Due to the triplet pregnancy, gaining extra 20 kg weight, distention of abdominal cavity, and increased intra-abdominal pressure, the patient was under the risk of worsening of angioedema. In literature, recommended dosage for prophylactic use of pdC1INH varies [Cinryze® Shire, FiercePharma, 1000 U intravenously (IV)] [11]; that is why pdC1INH dosages were increased up to 1,500 U every 2 days in our patient [12]. Moreover, there is a non-indication approval notice for prophylactic use in pregnancy which had been taken from the ministry of health in our country. This case has proven the safety and efficacy of pdC1INH despite the high dosages used, different from conventional treatment protocols. According to the study by Martinez-Saguer et al., in 83% of pregnancies, attack rates increased during pregnancy; highest mean rates were observed in the second and third trimesters [5]. The published review by Bouillet noted a close relationship between female hormones and angioedema [13]. In the context of bradykinin mediated angioedema, female sex hormones act on the kallikrein-kinin system by increasing synthesis of bradykinin. 17*β*-estradiol increases Hageman factor levels by stimulation of gene transcription and this hormone also increases kininogen and kallikrein levels [13]. Those results suggest that increased estrogenic status and kininogenase activities would have increased the frequency of abdominal attacks in our patient and this could have been prevented by the use of long term prophylaxis with pdC1INH. In another study, González-Quevedo et al. made a retrospective review of 61 C1-INH-HAE patients with 125 full-term pregnancies [14]. According to that study, pregnancy had a variable influence on the clinical expression of C1-INH-HAE. Although some of the patients reported an increased frequency of attacks, the others reported the presence of the same symptoms throughout pregnancy [14].

In summary, pdC1INH was effective as acute treatment, as well as for the long and short term prophylaxis of C1-INH-HAE during pregnancy and lactation. An important point in our case study is that the patient received estrogen raising therapies, especially* in vitro* fertilization methods like LHRH injections, without developing angioedema attacks. A triplet pregnancy was achieved safely without any increased attack rates by means of long term pdC1INH prophylaxis with the appropriate dosage according to her weight gain. The large cumulative dose of pdC1INH is safe for both the mother and the babies. Apparently, individualized treatment with administrations of different dosages of intravenous pdC1INH at different times can help physicians to manage C1-INH-HAE patients without complications despite increased estrogenic status. In conclusion, early discontinuation of attenuated androgens, regular monitoring of the patient, and the administration of intravenous pdC1INH in appropriate doses may be necessary during pregnancy and breastfeeding.

## Figures and Tables

**Table 1:** The schema of therapies.

Period	Therapy	Dosage and reasons of dosage changes	Attack frequency
Diagnosis (2004), 2013	Danazol peroral	50–200 mg/day (according to the patient's symptoms)	Abdominal attacks (every month before her menstrual cycle)

2013-2014 (*in vitro* fertilization therapies)	IV (intravenous) pdC1INH	1000 U/twice a week (LHRH injections were repeated four times. Each time LHRH was injected, and an extra dosage of 1000 U pdC1INH was administered)	No attack

2014-2015 (pregnancy < 20th week)	IV pdC1INH	1000 U every 2 days	No attack

2014-2015 (>20th week pregnancy-cesarean section)	IV pdC1-INH	1500 U every 2 days (She gained extra 20 kg weight) (Before and 12 hours after cesarean section, extra 1000 U pdC1INH was administered)	No attack

Postpartum period and breastfeeding (4 months)	IV pdC1INH	1000 U/twice a week (each abdominal attack was treated with extra 1000 U pdC1INH)	2 abdominal attacks
